# Economic and Diagnostic Biomarker Tests of Neonatal Sepsis: A Prospective Study from a Tertiary Care Hospital in a Low-Income Country

**DOI:** 10.1155/2022/5166380

**Published:** 2022-11-25

**Authors:** Priyatam Khadka, Govinda Maharjan, Ganesh Chapagain, JanukaThapaliya, Prakash Paudyal

**Affiliations:** ^1^Tribhuvan University, Tri-Chandra Multiple Campus, Kathmandu, Nepal; ^2^Nepal National Hospital, Kathmandu, Nepal

## Abstract

**Background:**

Neonatal sepsis is a leading cause of morbidity and mortality in low-and middle-income countries (LMICs). There are several sophisticated biomarkers; however, they are still insufficient in precision. In this perspective, our study aims to search for a pragmatic diagnostic biomarker in the age category.

**Methods:**

A cross-sectional study was conducted over six months(April-September 2018). All neonates with a diagnosis of probable sepsis were included. Logistic regression analysis of demographic variables was done to elucidate any association with confirmed sepsis cases. The median with interquartile range (IQR)] and mean with standard deviation (SD) were calculated, and then compared. The area under the receiver operating characteristic curve (AUROC) of the commonly opted biomarker tests [distribution width of red blood cells (RDW) and platelets(PDW), mean platelet volume(MPV), C-reactive protein (CRP), erythrocyte sedimentation rate (ESR)] was compared to the culture-confirmed case.

**Results:**

Of the 171 suspected sepsis subjects, we discovered a significant burden of newborn sepsis, with 18.7% of cases being culture-confirmed. 66 Early-onset sepsis(EOS) and 105 Late-onset sepsis(LOS) probable sepsis cases were enrolled. A higher incidence was revealed among male infants 24(14%) compared to females 8(4.7%). On logistic regression analysis, preterm birth [odds ratio (OR): 10.9, 95% confidence interval (CI): 4.5-26.9] and low birth weight (OR: 6.5, 95% CI: 2.4-17.9) were significantly associated. Coagulase-negative Staphylococcus aureus (CoNS) (n =6) among gram-positive, and *Pseudomonas aeruginosa* (n =6) was among gram-negative, were the leading etiologies. *Escherichia coli* (n =3) was the predominant bacteria in EOS subjects, while *Pseudomonas aeruginosa* (n =6) among LOS. Median interquartile range(IQR): platelet count 144.5(99-192), red cell distribution width 18(16.9-20), CRP 6(3-18.3); and mean ± SD: MPV (11.7 ± 1.7); PDW (15.2 ± 3.5) were attained, among confirmed cases. The AUROC, of biomarker tests was attained in the order: PDW(0.86) > MPV(0.81) > RDW(0.76) > CRP(0.67) > ESR(0.59); similarly, the cut-off order was >11.2, >10.4, >16.8, >2.9, >4.5, respectively.

**Conclusions:**

Our finding shows an increment in the width and volume of RBCand platelet: RDW, MPV, and PDW have a diagnostic role in neonatal sepsis.

## 1. Background

Sepsis is a leading cause of morbidity and mortality in neonates, accounting for one-third of all neonatal deaths worldwide [1]. However, the incidence and case fatality are distributed unevenly. The incidence of sepsis in high-income countries (HICs) ranges from 1 to 5 cases/1000 live births; however, it is estimated to be at least 3 to 20-fold higher in low- and middle-income countries (LMICs) [2–4]. Similarly, the case fatality rate in LMICs can reach up to 65%, while it is less than 2% in HICs [5, 6]. Mounting shreds of evidence have shown that the irony of neonatal sepsis is directly linked with existing healthcare facilities and the accessibility of early diagnostic modalities [3]. Probably, that could be the cause of the aforementioned discrepancies. Neonatal infection is one of the leading causes of hospital admissions and neonatal deaths in Nepal—the prevalence of the infection ranges between 2 and 4% [7]. In 2015, 12,881 neonatal deaths were reported in Nepal, of which 18.4% were attributed to sepsis [6]. The data could be even worse since numerous undocumented neonatal deaths are yet to be enrolled in Nepal's National Database due to deficient recording, reporting, and data analyzing modalities.

For early diagnosis, a myriad of sophisticated biomarker tests are available; however, they are still insufficient in terms of precision and accessibility. Turning to LMICs, the cost efficiency and accessibility of diagnostic tests are another paramount concern. Therefore, cost-efficient, reliable, and early predictors/biomarkers of neonatal sepsis are being searched for worldwide. With this backdrop, our study aims to search for pragmatic biomarker tests for neonatal sepsis.

## 2. Methods

### 2.1. Design of the Study and the Population Sample

A cross-sectional study was conducted at Nepal National Hospital (NNH), a multi-speciality tertiary care hospital in Nepal, for 6 months (April-September 2018). All neonates admitted to the NICU and PICU with a presumptive clinical diagnosis of sepsis were enrolled. A pre-tested questionnaire was administered to each suspected neonate and their parents were approached for consent to take part in the study (Supplementary Material 1). Data regarding personal information (patient's demographics, including residence locality) and existing infectious diseases were coded and kept confidential.

### 2.2. Inclusion and Exclusion Criteria

All clinically suspected and consented neonates admitted to NNH during our study period were included in the study. However, neonates presenting with major congenital abnormalities and those previously treated as sepsis cases were excluded from the study. The categorization of congenital abnormalities in our study was based on the European Surveillance of Congenital Anomalies (EUROCAT) guidelines [8].

### 2.3. Diagnosis of Sepsis

The clinical investigation/suspicion of sepsis was made by a unit pediatrician relying upon the clinical history, likely occurring clinical signs, and/or underlying risk factors. As per the NICU protocol followed by Nepal National Hospital, sepsis was defined as culture-positive sepsis(confirmed sepsis) if the blood culture was positive in a patient with clinical signs of sepsis. However, a negative blood culture with existing clinical signs was assessed as culture-negative sepsis (probable sepsis). In reference to WHO classification, we considered babies born alive before 37 weeks of pregnancy as preterm neonates. However, further characterization of extremely preterm(less than 28 weeks), very preterm (28 to 32 weeks), and moderate to late preterm(32 to 37 weeks) was not done in our study. Likewise, babies born alive after the completion of 37 weeks of pregnancy were defined as full-term neonates.

### 2.4. Microbiological Investigation

Neonatal sepsis could be caused by one or more systemic infections such as septicemia, urinary tract infections, pneumonia, meningitis, arthritis, or osteomyelitis; nevertheless, neonates with positive blood culture results were considered confirmed sepsis cases [9].

1-2 ml of peripheral blood samples were drawn from all neonates, those having a presumptive diagnosis of sepsis. Aseptically, the samples were injected into BD-BACTEC Peds Plus/F culture vials (Becton Dickinson, UK) and incubated in an automated BD BACTEC FX40 (Becton Dickinson, UK) culture system. Upon an indication of culture-positive, a small drop (approximately a loopful volume) of blood was aspirated and inoculated on 5% sheep blood agar, MacConkey agar, and chocolate agar. Further, isolation and identification of the isolates were done by standard microbiological techniques—biotyping (colony morphology, staining reaction, and biochemical characteristics) and serotyping using specific antisera(Denka Seiken Co. Ltd., Tokyo, Japan). The antimicrobial susceptibility testing (AST) was performed by the disk diffusion method [modified Kirby-Bauer method] on Mueller Hinton agar (Hi-Media, India) in compliance with standard procedures recommended by the Clinical and Laboratory Standards Institute (CLSI), Wayne, PA, USA [10].

### 2.5. Serological and Hematological Indicators of Sepsis

Quantitative serum CRP level was measured by an automatic clinical chemistry analyzer –XL 200 ERBA Diagnostic Mannheim. The Sysmex (XS-500i) automated hematology system analyzer was used to measure complete blood cell (CBC) parameters (Total leukocytes count, Hemoglobin, Hematocrit, Platelet count, Mean Platelet Volume (MPV), Platelet Distribution Width (PDW), Plateletcrit (PCT), Red Blood Cell (RBC), Mean Corpuscular Volume (MCV), Mean Corpuscular Haemoglobin (MCH), Mean Corpuscular Haemoglobin Concentration(MCHC). ESR(erythrocyte sedimentation rate) was determined with standardized methods of miro-ESR. Blood was collected in a pre-heparinized 75 mm long micro-hematocrit tube (Fisher brandTM micro-hematocrit Capillary Tubes) with an internal diameter of 1.1 mm and an external diameter of 1.5 mm and allowed to stand vertically for an hour. After 1 hour, a fall in the column of blood in the capillary tube was measured.

### 2.6. Data Analysis

Statistical analysis was conducted in SPSS (version 20.0). All qualitative variables were expressed in absolute frequency(N) with percentage(%), while quantitative variables were calculated as mean ± standard deviation (SD) or median with interquartile range(IQR). Logistic regression analysis of demographic variables was done to elucidate any association with confirmed sepsis cases; an odds ratio with 95% confidence interval(CI) was calculated. Receiver Operating Characteristics (ROC) curve analysis was done to evaluate the utility and area under ROC curve (AUROC) was compared. *P* value≤0.05 was considered a significant threshold in these statistical evaluations.

## 3. Results

### 3.1. Patients' Demographic Features

In this cross-sectional investigation, we discovered a high burden of newborn sepsis, with 18.7% of 171 suspected sepsis individuals having the condition. The confirmed sepsis rate was higher in males 24(14%) compared to females 8(4.7%). LOS occurred in 23 (13.5%) of the culture-positive sepsis events. Among demographic variables, preterm delivery and low birth weight were significantly associated with sepsis. On logistic regression analysis, preterm birth [odds ratio (OR): 10.9, 95% confidence interval (CI): 4.5-26.9] and low birth-weight (OR: 6.5, 95% CI: 2.4-17.9) were assessed (Table 1).

### 3.2. Microbiological Findings: Distribution of Sepsis-Causing Isolates

Coagulase-negative *Staphylococcus aureus*(CoNS) (n =6) among gram-positive bacteria(GPB), and *Pseudomonas aeruginosa* (n =6) among gram-negative bacteria(GNB), were the leading etiologies in our study. *Escherichia coli* (n =3) was the predominant bacteria in EOS subjects, while *Pseudomonas aeruginosa* (n =6) among LOS (Table 2).

### 3.3. Laboratory Findings: Biomarker Tests and their Comparisons in Neonatal Sepsis

**Figure 1:**
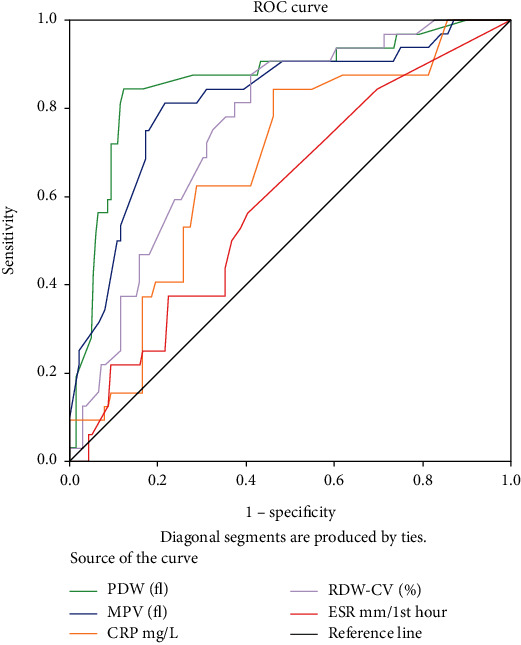
ROC curve.

Among culture-confirmed cases, Median IQR: platelet count 144.5(99-192), red cell distribution width 18(16.9-20), CRP 6(3-18.3); and mean ± SD: MPV (11.7 ± 1.7); PDW (15.2 ± 3.5) were attained (Table 3). ROC curve was used to compare the test efficacy of implicated diagnostic parameters to sepsis, viz. PDW, MPV, CRP, RDW-CV, and ESR (Figure 1). The AUROC of biomarker tests was attained in the order: PDW(0.86) > MPV(0.81) > RDW(0.76) > CRP(0.67) > ESR(0.59); similarly, the cut-off order was >11.2, >10.4, >16.8, >2.9, >4.5, respectively (Table 4).

## 4. Discussion

In this cross-sectional study, we found a high burden of neonatal sepsis: of 171 probable sepsis subjects, 32(18.7%) were found to be culture-confirmed sepsis. Neonates with preterm delivery and low birth weight are the demographic variables significantly associated with sepsis. Sepsis in preterm infants remains a significant clinical problem which is further complicated by disease heterogeneity and the absence of rapid and reliable diagnostic tests [11, 12]. Our study tries to address these diagnostic complexities. Relying on the gold standard diagnostic culture, this study demonstrated 9.9% of the preterm neonates had suffered from sepsis. The finding is nearly half of the corresponding findings of Jain et al. 20-40% [13] and Salma et al. 21.5% [11]. The deficient immune system, compromised innate immune system, and an increased need to support invasive devices are commonly cited risk factors for sepsis among preterm neonates [12].

Geographical variations have been observed in the incidence of neonatal sepsis. In South Asia, the variable prevalence of culture-positive sepsis was reported to be from 6 to 57% [4, 14–17]. Although this study's rate of culture-proven sepsis (18.7%) is less than half that of the surrounding studies' reported 35.5% rate [18]. Furthermore, similar studies conducted in other developing countries from Africa and Asia have reported: Ethiopia(44.7%), Uganda(37%), Nigeria(45.9%), Ghana(17%), Tanzania(47.1%), Bangladesh(34.9%) and India(25 to 45%) [5, 19–24]. This is attributed to changing patterns of antibiotic use and lifestyles [25]. On a specific note, culture-negative does not preclude sepsis because nearly 26% of neonatal sepsis could be due to anaerobic organisms. Additionally, viruses(rubella, cytomegalovirus), protozoans (*Toxoplasma gondii*), and treponema (*treponema pallidum*) have also shown their possible implications [25, 26]. Therefore, a diagnosis of consideration is required.

The epidemiology of neonatal sepsis has been found to change with time. Since 1990, EOS has decreased significantly; however, the LOS has remained unchanged [27, 28]. In our study, the proportion of LOS was higher than EOS (LOS13.5% vs. EOS 5.3%), similar to the aforementioned studies [18, 23, 29]. Of culture-proven sepsis, nearly 72% of episodes are late-onset types, suggesting the horizontal transfer of infection in our study; the finding is comparable to the other Asian countries [30, 31]. However, in other Asian countries such as Pakistan and Bangladesh, the rate of vertical transmission, i.e. the early-onset type was found to be higher [15, 32]. Poor hygienic procedures during delivery were believed to be the reason behind this upset.

The individualized isolates contributing to sepsis could be different due to diverse patients' residences, hygienic practices, and hospital settings/protocols implicated in infection control. In this study, GNB was the leading cause of both early and late-onset sepsis, which corresponds to elucidations by several research institutes of LMICs [4, 33, 34]. The predominant isolates contributing to sepsis were *Pseudomonas aeruginosa* followed by *Escherichia coli* in our study; nevertheless, the nearby hospital reported *Klebsiella pneumonia* as the commonest cause of sepsis [4, 18, 35, 36]. Turning to GPB, CoNS followed by *Staphylococcus aureus* was the commonest pathogen behind neonatal sepsis in our study; the finding is similar to that reported by Manandhar et al. [4] and Sahet. al [18], .from the neighboring hospitals. Nonetheless, the bacterial predominance was found to be reversed in another similar study [36].

The comparison of commonly employed, accessible, and cost-effective biomarker tests against neonatal sepsis is crucial—particularly for those people who reside in low-income countries. The illustrative findings could assist in policymaking and designing strategic outlines. With this rationale, we conducted this study. Complete blood cells, including RDW and PDW, CRP, and ESR were compared to the gold standard bacterial culture.

The usefulness of CBC as a biomarker of neonatal sepsis has not yet been proven due to poor positive and negative predictive values; however, the importance of a normal CBC value in ruling out sepsis should not be underestimated [37–42]. Several studies have shown that total leukocyte count, platelet count, absolute neutrophil count, red cell indices, and the immature-to-total neutrophil ratio correlate with neonatal sepsis [37, 41–43]. In our study, platelet count, MPV, and MCH have shown a significant association with neonatal sepsis. These findings are consistent with some relevant kinds of literature [44–49].

Sepsis has been shown in clinical and experimental studies to have a wide range of effects on erythrocytes—altering the heterogeneity of RDW, though its diagnostic implications are still being debated [50]. Therefore, we tried to explore the diagnostic application of RDW in our study. In our study population, RDW was significantly higher in confirmed sepsis cases with a median IQR =18(16.9-20) with a *P-value:*<0.001. The finding is in line, as reported by Hu et al., who reported 18.9-19.9% as the diagnostic threshold for neonatal sepsis [51]. Similar other studies support our hypothesis on the diagnostic values of RDW in neonatal sepsis [52–56].

Several diagnostic models have supported PDW as an early and economic biomarker of sepsis. While testing the applicability of PDW in sepsis diagnosis, we observed PDW in culture-confirmed sepsis was 15.2 ± 3.5 while in probable sepsis it was 10.5 ± 3.09. The illustration is in line with the findings of Ahmad et al. as he reported a median PDW 12.3 in confirmed sepsis cases [57]. Majumdar et al. and Patrick et al. reported increased PDW by >19.1% among neonates has the diagnostic value, however [58, 59].

Platelet indices (platelet count, MPV, PCT, and immature platelet fraction) are acute phase reactants and these parameters could vary with inflammation and increased platelet consumption/production [60]. Pieces of evidence have shown that platelet indices could be both predictive as well as prognostic biomarkers in neonatal sepsis [60, 61]. In the present study, we compared the platelet indices (platelet count, MPV, and PCT) as predictive biomarkers in neonatal sepsis cases. In confirmed sepsis cases, Median (IQR) platelet count: 144.5(99-192); mean ± SD, MPV: 11.7 ± 1.7; mean ± SD, PCT%: 0.20 ± 1.15 were observed. Of them, platelet count and MPV were statistically significant. Our findings are well-supported by the conclusions of Majumdar et al. [58], Mishra S et al. [60], and Dundar B et al. [61].

C-reactive protein, an infectious inflammatory biomarker, may complement the evaluation of clinical signs and risk factors within the diagnosis of sepsis—several studies have shown its applicability in precocious diagnosis as the test is easily available [62–64]. We found the median serum CRP was significantly higher in confirmed sepsis cases compared to probable sepsis, being 6 mg/L(IQR =3-18.3) in confirmed sepsis and 2.7 mg/L (IQR =2.1-7.7) in probable sepsis cases. The assertion of Tosson et al. is in line with our findings [65]. Earlier, in a study from Nepal, Shah et al. illustrated the sensitivity (44%) and specificity (92%) of CRP among sepsis proven-cases which supports its validity as a biomarker test [66]. However, diagnosis of consideration is required: the spurious increase in the level of CRP in numerous conditions such as meconium aspiration syndrome, delayed transition after birth, premature infant exposure to glucocorticoids, prolonged rupture of membranes, stressful delivery or fetal distress, prolonged labor, maternal fever during labor, perinatal asphyxia or shock, surfactant administration, intra-ventricular hemorrhage, and pneumothorax, may mislead the diagnosis [37, 64, 67].

Micro-erythrocyte sedimentation rate is single, cheap, easy to perform, and less time-consuming with a sensitivity of 63.3% and 60%, respectively [68]. In a resource-limited setting, as a supportive biomarker, ESR is often used as a diagnostic tool. Therefore, we added the test to our study. In our study, the median micro-ESR was 6.5(IQR =3-14); although the statistical association was not found as an alternative in diagnosis and in monitoring therapeutic progress [69, 70]. Similar studies done in Nepal and India have shown the significance of micro-ESR levels concerning confirmed sepsis: Manandhar et al. from Nepal found 17.3% of culture-confirmed sepsis had elevated ESR by 33.3%, while Ghaliyaz et al. from India found 32% of culture-confirmed sepsis had elevated ESR by 38% [70, 71].

These economical and commonly accessible biomarker tests were compared with ROC curve analysis. The AUROC of the biomarker test was attained in the order: PDW(0.86) > MPV(0.81) > RDW(0.76) > CRP(0.67) > ESR(0.59); similarly, the cut-off order was >11.2, >10.4, >16.8, >2.9, >4.5, respectively. The diagnostic applicability of these biomarker tests in neonatal sepsis has not been compared before.

## 5. Limitations

The broad range of other biomarker tests could not be compared because of deficient funding and starved laboratory settings—only tests available in our setting were compared.

## 6. Conclusion

Particularly in LMICs, the comparison of accessible and cost-effective biomarker tests in the diagnosis of neonatal sepsis is crucial since healthcare facilities are not approachable to everyone. Our study revealed that the increment in the width and volume of RBC and platelets has a promising role in the diagnosis of neonatal sepsis. Therefore, these could assist in policy making and designing diagnostic outlines for neonatal sepsis. Validation with larger prospective studies on these biomarker tests is required, however.

## Figures and Tables

**Table 1:** Neonatal demographic characteristics.

Patient demographics	Culture confirmed sepsis (%)	Culture negative sepsis (%)	Total (%)	Odds ratio	95% CI	P value
*Gender*						
Male	24 (14)	84 (49.1)	108 (63.2)	1.96	0.82-4.68	0.15
Female	8 (4.7)	55 (32.3)	63 (36.8)			
*Delivery type*						
Preterm	17 (9.9)	13 (7.6)	30 (17.5)	10.9	4.5-26.9	<0.001
Full-term	15 (8.8)	126 (73.7	141 (82.5)			
*Weight by birth*						
Low birth weight	10 (5.8)	9 (5.3)	19 (11.1)	6.5	2.4-17.9	<0.001
Normal birth weight	22 (12.9)	130 (76.0)	152 (88.9)			
*Sepsis type suspected*						
Early-onset	9 (5.3)	57 (33.3)	66 (38.6)	0.5	0.24-1.3	0.22
Late-onset	23 (13.5)	82 (48)	105 (61.4)			

Gestational age in weeks (Preterm): Mean with SD =31.8 ± 3.33; Median with IQR =33(34.7-28.25).

**Table 2:** Distribution of isolated organism.

Etiologies of probable sepsis	Early-onset	Late-onset	Total (%)
No growth	57	83	140(81.9)
Gram negative bacteria			
*Escherichia coli*	3	2	5(2.9)
*Klebsiella pneumonia*	0	3	3(1.8)
*Pseudomonas aeruginosa*	0	6	6(3.5)
*Acinetobacter baumanii-complex*	0	1	1(0.6)
*Citrobacter freundii*	1	1	2(1.2)
*Burkholderia cepacia*	0	1	1(0.6)
Gram positive bacteria			
*Coagulase negative staphylococcus*	2	4	6(3.5)
*Staphylococcus aureus*	2	2	4(2.3)
*Enterococcus fecalis*	1	2	3(1.8)
Total (%)	66(38.6%)	105(61.4%)	171(100)

**Table 3:** Laboratory findings among probable-sepsis-neonates.

Biomarker test	Confirmed sepsis	Probable sepsis	P value
WBC (10^3*μ*L) median (IQR)	13.9 (10.2-16.1)	11.7 (8.3-15.05)	0.53
HB (g/dl) mean ± SD	15.3 ± 2.4	15.2 ± 2.4	0.8
Hematocrit (%) mean ± SD	48.7 ± 13.6	45.6 ± 7.1	0.06
Platelet count(10^3*μ*L) median (IQR)	144.5 (99-192)	249 (199-312)	0.001
MPV (fl) mean ± SD	11.7 ± 1.7	9.6 ± 1.7	<0.001
PDW (fl) mean ± SD	15.2 ± 3.5	10.5 ± 3.09	<0.001
PCT (%) mean ± SD	0.20 ± 1.15	0.25 ± 0.12	0.06
RBC (10^6*μ*L) mean ± SD	4.9 ± 0.8	4.7 ± 0.7	0.4
MCV (fl) mean ± SD	93.8 ± 6.08	95.4 ± 5.8	0.1
MCH (pg) mean ± SD	33.07 ± 1.3	33.4 ± 1.3	0.04
MCHC (%) mean ± SD	33.07 ± 1.1.36	33.4 ± 1.3	0.14
RDW-CV (%) median (IQR)	18 (16.9-20)	15.6 (11.5-17)	<0.001
ESR (mm/1st hour) median (IQR)	6.5 (3-14)	4 (2-12)	0.078
CRP(mg/L) median (IQR)	6 (3-18.3)	2.7 (2.1-7.7)	0.045

**Table 4:** Area Under ROC curve.

Test result variable(s)	Area	Std. Error^a^	Asymptotic sig.^b^	Asymptotic 95% confidence interval	
				Lower bound	Upper bound
PDW(fl)	.862	.040	.001	.784	.941
MPV (fl)	.816	.044	.001	.730	.903
CRP mg/L	.675	.050	.002	.577	.772
RDW-CV (%)	.760	.042	.001	.678	.842
ESR mm/1st hour	.596	.054	.092	.490	.701

The test result variable(s): PDW (fl), MPV (fl), CRP mg/L, RDW-CV (%), ESR mm/1st hour has at least one tie between the positive actual state group and the negative actual state group. Statistics may be biased. a. Under the nonparametric assumption. b. Null hypothesis: true area =0.5.

## Data Availability

The data generated or analyzed during this study are included in this published article, and the remainder is available upon reasonable request from the corresponding author.

## Abbreviations

AUROC: Area under receiver operating characteristics
CBC: Complete blood cell
CRP: C-reactive protein
EOS: Early-onset sepsis
ESR: Erythrocyte sedimentation rate
GNB: Gram-negative bacteria
GPB: Gram-positive bacteria
LOS: Late-onset sepsis
LMICs: Low-and middle-income countries
PCT: Plateletcrit
ROC: Receiver operating characteristics.

## Supplementary Materials

Supplementary Materials Supplementary Material. Questionaire for demographic features.
