# Supplementary material for: Economic and Diagnostic Biomarker Tests of Neonatal Sepsis: A Prospective Study from a Tertiary Care Hospital in a Low-Income Country
Source: Biomed Res Int. 2022 Nov 25;2022:5166380. doi: 10.1155/2022/5166380 (PMC9718631; doi:10.1155/2022/5166380)
Supplement: Supplementary Materials — Supplementary Material. Questionaire for demographic features. [file 5166380.f1.docx]

| **Questionnaire** | | | | | | | | |  |
| --- | --- | --- | --- | --- | --- | --- | --- | --- | --- |
|  |  |  |  |  |  |  |  |  |  |
|  |  |  |  |  |  |  |  |  |  |
| Patient's ID. | |  | Age/Sex: |  |  | Address: |  |  |  |
|  |  |  |  |  |  |  |  |  |  |
|  |  |  |  |  |  |  |  |  |  |
| Relation with patient: ……………... | |  | Guardian profession: ………….. | | | Observed financial status……………… | | |  |
|  |  |  |  |  |  |  |  |  |  |
|  |  |  |  |  |  |  |  |  |  |
| In-patient/Out-patient: ………………. | | |  |  | Religion: …………. |  |  |  |  |
| Unit/Ward…... |  |  |  |  |  |  |  |  |  |
|  |  |  |  |  |  |  |  |  |  |
| Previous history of antimicrobial therapy (if any) | | | | ………………………………………………………………………………………. | | | | |  |
|  |  |  |  |  |  |  |  |  |  |
|  |  |  |  |  |  |  |  |  |  |
| Previously admitted(Y/N) | | …………………. |  |  | If admitted: | Duration of stay | | …………………… |  |
|  | | | |  |  | Previous diagnosis | | ………………….. | |
| Continental abnormalities(Y/N) | | | …………….. |  |  | Any surgery? (Y/N) | | …………………… |  |
|  |  |  |  |  |  |  |  |  |  |
|  |  |  |  |  |  |  |  |  |  |
| Delivery type |  | Pre-term | …………….. |  | Weight by birth(Kg): | | ……………… | |  |
|  |  | Full-term | …………….. |  |  |  |  |  |  |
|  |  |  |  |  |  |  |  |  |  |
|  |  |  |  |  |  |  |  |  |  |
| Observed symptoms : | | ……………………………………….. | |  |  |  |  |  |  |
|  |  | ……………………………………….. | |  |  |  |  |  |  |
|  |  | ……………………………………….. | |  |  |  |  |  |  |
|  |  | ……………………………………….. | |  |  |  |  |  |  |
| **The content of the information sheet that was provided have been read carefully by me/explained in detail to me, in a language that I comprehend, and I have fully understood the contents. I confirm that I have had the opportunity to ask questions.** | | | | | | | | | |
|  |  |  |  |  |  |  |  |  |  |
| …………………… |  |  |  |  |  | ………………………………. | | |  |
| Legal Parent's signature /Date | | |  |  |  | Researcher signature/Date | | |  |
